# Sustainable Polyhydroxyalkanoate Production from Food Waste via *Bacillus mycoides* ICRI89: Enhanced 3D Printing with Poly (Methyl Methacrylate) Blend

**DOI:** 10.3390/polym15204173

**Published:** 2023-10-20

**Authors:** Marian Rofeal, Fady Abdelmalek, Joanna Pietrasik

**Affiliations:** 1 International Center for Research on Innovative Biobased Materials (ICRI-BioM)—International Research Agenda, Lodz University of Technology, Zeromskiego 116, 90-924 Lodz, Poland; 2 Department of Botany and Microbiology, Faculty of Science, Alexandria University, Alexandria 21521, Egypt; 3 Chemical Engineering Department, Polytechnique Montreal, Montreal, QC H3T 1J4, Canada; 4 Department of Engineering Physics, Polytechnique Montreal, Montreal, QC H3T 1J4, Canada; 5 Faculty of Chemistry, Institute of Polymer and Dye Technology, Lodz University of Technology, Stefanowskiego 16, 90-537 Lodz, Poland; joanna.pietrasik@p.lodz.pl

**Keywords:** PHB, PMMA, 3D printing, food waste, biopolymers, biodegradable

## Abstract

In view of implementing green technologies for bioplastic turning polices, novel durable feedstock for *Bacillus mycoides* ICRI89 used for efficient polyhydroxybutyrate (PHB) generation is proposed herein. First, two food waste (FW) pretreatment methods were compared, where the ultrasonication approach for 7 min was effective in easing the following enzymatic action. After treatment with a mixture of cellulase/amylases, an impressive 25.3 ± 0.22 g/L of glucose was liberated per 50 g of FW. Furthermore, a notable 2.11 ± 0.06 g/L PHB and 3.56 ± 0.11 g/L cell dry eight (CDW) over 120 h were generated, representing a productivity percentage of 59.3 wt% using 25% FW hydrolysate. The blend of polyhydroxybutyrate/poly (methyl methacrylate) (PHB/PMMA = 1:2) possessed the most satisfactory mechanical properties. For the first time, PHB was chemically crosslinked with PMMA using dicumyl peroxide (DCP), where a concentration of 0.3 wt% had a considerable effect on increasing the mechanical stability of the blend. FTIR analysis confirmed the molecular interaction between PHB and PMMA showing a modest expansion of the C=O stretching vibration at 1725 cm^−1^. The DCP-PHB/PMMA blend had significant thermal stability and biodegradation profiles comparable to those of the main constituent polymers. More importantly, a 3-Dimetional (3D) filament was successfully extruded with a diameter of 1.75 mm, where no blockages or air bubbles were noticed via SEM. A new PHB/PMMA “key of life” 3D model has been printed with a filling percentage of 60% and a short printing time of 19.2 min. To conclude, high-performance polymeric 3D models have been fabricated to meet the pressing demands for future applications of sustainable polymers.

## 1. Introduction

The production of a large amount of synthetic plastics has been driven by global needs in various sectors such as the food packaging industry, household items, and the biomedical sector. In addition, the utilisation of one-use plastics has dramatically increased after the COVID-19 crisis in terms of the consumption o surgical gloves and face masks, reaching about 69 billion units per month [1]. Several global investigations have recently reported that more than 150 million tons of plastics have been dumped in oceans, less than 10% of which could be potentially biodegraded [2,3]. Scientists are therefore urged to come up with more practical and economic strategies for the large-scale production of sustainable bioplastics. Biodegradable plastics are deemed promising substitutes for petroleum-based sources owing to their eco-friendliness, biodegradation, and safety [4,5]. Polyhydroxybutyrate (PHB) is the simplest homopolymer of polyhydroxyalkanoates (PHAs), which can be produced via a wide range of bacterial species, such as *Bacillus* sp. [6], *Rhodococcus* sp. [7], *Cupriavidus* sp. [8], *Pseudomonas* sp. [9], and others [10]. However, the high cost of substrates and cultures is still hindering huge manufacturing streams. In fact, substrate expenditure alone comprises more than approximately 40% of the whole process, which makes it nine times more expensive than non-biodegradable plastics [11].

For this reason, there has been a clear shift toward the effective bioconversion of natural resources, especially the valorisation of food waste (FW). Owing to being ubiquitous and the possibility of significantly reducing waste when bioconverted to bioplastics, FW is a promising candidate for providing cost-effective carbon sources [12]. In a recent experiment, discarded FW that is rich in rice, noodles, meat, and vegetables was repurposed as an eco-friendly resource for producing PHA using laboratory-scale bioreactors operating in a fed-batch mode, with *Haloferax mediterranei* as the microorganism of choice [13]. In the same context, a pilot plant employed mixed microbial cultures for a continuous period of 6 months, utilising locally sourced FW as the primary feedstock. This process yielded an impressive PHA content of 47.91 ± 1.91% [14]. According to each food supply composition, its management techniques differ in terms of upstream processing, fermentation conditions, and microbial producers. FW usually consists of cellulose, starch, lipids, fibers, proteins, and other components. Organic FW, in particular, is too complicated for microbial metabolism to be used directly [15]. In this context, pretreatment methods are fundamental to transforming FW into PHB precursors. Soundwaves with a frequency of 20 kHz or higher are referred to as ultrasound. Through acoustic cavitation, ultrasound may produce strong shear forces that can mix fluids, speed up mass transfer, and disperse particles. Furthermore, ultrasound has been used to prepare different wastes for enzymatic hydrolysis later [16]. It has been demonstrated that ultrasonic pretreatment works well in promoting the enzymatic hydrolysis of starchy food crops. However, its usage in the bioconversion of FW has not been extensively studied. Recently, Pau et al. recommended the ultrasonication (680 W) of cooked FW for 10 s with the aim of enhancing lactic acid production via commercial *Lactobacillus* sp. [17]. Furthermore, the particle size can be decreased with the use of sonication, increasing the surface area susceptible to enzymatic attack. For example, a mixture of amylolytic and cellulolytic formulations has effectively hydrolysed FW pretreated with sono-electrochemicals, freeing 17.3 g of glucose/100 g of FW [18].

Another avenue is 3-dimensional (3D) printing, which is a revolutionary approach that enables the quick creation of a physical prototype from a virtual idea using a 3D computer-aided design. Even with a very complicated model, this method may reduce production time by up to 50% [19]. However, PHB has some shortcomings curtailing its applicability in 3D printing, such as high fragility, low elongation at break (EB), and low thermal stability, with a degradation temperature of around 200 °C [20]. Furthermore, the 3D printing of such crystalline polymers usually brings about poor dimensional precision or distortion in production. Biopolymers are usually retained at the melting temperature throughout the printing process, and they should stay stable without altering their properties.

In this regard, blending with other polymers, including synthetic or biopolymers, has been proven to address these drawbacks for better processability and industrialisation [21]. Among the various polymers investigated, poly(methyl methacrylate) (PMMA) is one of the most common thermoplastics to be mixed with biopolymers due to its high thermal stability, environmental inertness, and powerful ability to form strong molecular interactions with biopolymers [22,23]. In a previous study, PMMA demonstrated high reactivity with poly(lactic acid) (PLA) in the presence of poly (styrene-co-glycidyl methacrylate) copolymer, where PMMA could overcome PLA brittleness. PLA/PMMA blends (80/20 wt%) exhibited ductile behaviour, with a significant improvement in EB and impact resistance compared to neat polymers [24]. From another perspective, chemical crosslinking is a peroxide-based method of introducing crosslinking structures into polymers. Peroxide-induced crosslinking is a common process for producing high-performance polymeric blends. Thus, in situ reactive compatibilisation can enhance characteristics since it is an efficient, fast, solvent-free, low-cost, and environmentally friendly technique for processing polyester blends [25]. Previously, dicumyl peroxide (DCP) was utilised to make PLA polymeric mixtures compatible. PLA/poly(butylene adipate-co-terephthalate) was compatibilised with 0.2% DCP, resulting in higher tensile, ductile, and impact strength [26]. Therefore, we hypothesised that due to the ability of DCP to enhance the mechanical and thermal performance of the polymeric blends, it could be a powerful tool for constructing reliable 3D platforms.

The current investigation has two main objectives. The first is to present a successful endeavour for adequate FW hydrolysate saccharification into fermentable sugars to support PHB generation. Ultrasonication and organosolv pretreatment methods were compared for a better glucose yield. An enzymatic mixture was tailored for potent FW hydrolysis. Then, a series of PHB/PMMA blends were prepared and characterised. The effect of DCP peroxide radicals on the developed blends was examined in terms of mechanical properties, thermal stability, and biodegradability. We aimed to establish a mild crosslinked polymeric matrix for better printing processability. Finally, the 3D printing capacity of the obtained matrix was investigated to propose economical and eco-friendly thermoplastic products for durable application. To date, this is the first report that presents FW hydrolysate to be used by *Bacillus mycoides* ICRI89 for sustainable PHB biosynthesis. Moreover, no previous studies have examined the effect of DCP crosslinking on PHB/PMMA blends, generating high-performance 3D models.

## 2. Materials and Methods

### 2.1. Materials and Chemicals

Commercial cellulase enzyme (NewCell Conc L) was purchased from NewEnzymes, Pedrouços, Portugal. Cellulase and 2-benzisothiazolin-3(2H)-one (>1%) made up the enzyme preparation. α-Amylase from *Bacillus* sp., type II-A, lyophilised powder (≥1500 units/mg protein), glucoamylase from *Aspergillus niger*, powder (~120 U/mg), poly (methyl methacrylate) (Mw: 50,000 Da), Lipase B from *Candida antarctica* immobilised on Immobead 150, recombinant from *Aspergillus oryzae* with enzyme activity ≥1800 U/g, and dicumyl peroxide (DCP) were provided from Sigma-Aldrich, Darmstadt, Germany. All chemicals for the microbial cultures were obtained from Pol-Aura, Olsztyn, Poland. The other chemicals and solvents were purchased from Alchem, Wrocław, Poland.

### 2.2. Food Waste Collection

Food waste (FW) was collected from restaurants on the campus of Lodz University of Technology in Lodz, Poland. Plastic, paper, towels, and glass that might be recycled were physically removed from the FW. The organic part of the FW was made up of waste rice, pasta, bread, fruit peels and skins, vegetable residues, and chicken and pork meat residues. The FW was air-dried for two weeks before being ground in a grinder (IKA Benchtop A 10 Basic Mill, Staufen, Germany). Finally, the FW powder was vacuum-packed in plastic bags for later usage. The FW powder contained 68.5% starch, 25.9% lignocellulose, and 5.6% other components including lipids, proteins, and pectin. The methods of McCleary et al. and Van Soert and Robertson were applied to determine the content of the FW powder [27,28].

### 2.3. Pretreatment of Food Waste

The FW pretreatment before enzymatic hydrolysis was performed by either sonication or ethanolic organosolv methods.

#### 2.3.1. Ultrasonic Pretreatment of FW

To prevent starch gelatinisation, FW powder was combined with 0.05 M citrate buffer (pH 5.5) to produce slurries in a solid-to-liquid ratio of 5% *w*/*v*. A bench-scale 20 kHz ultrasonic device (Branson digital sonifier 450-D, Danbury, CT, USA) was used to sonicate the FW slurries. An ultrasonic horn (25 mm) with a power input of (0.8 W/mL) was placed into the FW samples to execute the ultrasonic pretreatment for different time intervals of 3, 5, 7 and 10 min at a constant temperature of 25 °C [29].

#### 2.3.2. Ethanolic Organosolv Pretreatment of FW

The ethanolic organosolv pretreatment was carried out at 120 °C for 30, 60, and 120 min using an ethanol–water solution (80% *v*/*v*) containing a pretreatment catalyst; acetic acid (1.5% *w*/*w* FW dry weight). The pretreatment was conducted in a 500 mL high-pressure stainless-steel cylinder container equipped with a thermometer and a pressure gauge. After 10 g of dry FW and 190 mL of the pretreatment solution were added, the reaction container was heated in an oil bath. The reactor was cooled to room temperature in an ice bath after pretreatment. Vacuum filtration was used to remove the pretreated particles from the pretreatment solution, and then the solids were washed with a 300 mL solution of 80% ethanol at 60 °C. The pretreated FW solids were then rinsed with distilled water and allowed to air-dry for 48 h. Plastic bags were used to store dried, pretreated FW for enzymatic hydrolysis [30].

### 2.4. FW Enzymatic Hydrolysis

Both FW pretreatment with sonication and ethanolic organosolv methods were enzymatically hydrolysed, searching for the highest glucose release. Since the pretreated FW is mainly composed of two fractions, starch and cellulose, there were two successive enzymatic hydrolysis steps. Initially, the starch fraction was targeted by introducing 0.1 g/g FW of α- amylase and glucoamylase individually. To illustrate, 50 g FW biomass was added to 1000 mL of 0.05 M sodium citrate buffer (pH 4.8) and autoclaved at 121 °C for 20 min. Then, CaCl_2_ (1 g/L) was added to the mixture as a catalyst. For starch liquefaction, the conditions of the mixture were adjusted to be pH 6, 90 °C, for 2 h. Following this, starch saccharification was conducted with glucoamylase by adjusting the mixture conditions at pH 4.8, 65 °C, for 24 h [31].

Subsequently, cellulase enzyme with a concentration of 1.5% (*v*/*v*) and specific activity of 65 ± 0.3 FPU/mL and enzyme protein with a concentration of 122 ± 0.3 mg/mL were added to the mixture to digest the cellulosic portion of the pretreated FW at pH 4.8, 45 °C, 120 rpm, for 72 h. After hydrolysis, the remaining solids were separated from the liquid fraction via centrifugation at 5000 rpm for 10 min. Samples of the hydrolysates were withdrawn aseptically using sterile syringes for glucose analysis. A glucose colourimetric kit (Biomaxima, Lublin, Poland) was used to quantify the glucose release of hydrolysed liquors [11]. The pretreatment method resulting in the highest glucose release after enzymatic hydrolysis was selected and proposed for a sustainable fermentation process.

### 2.5. PHB Production Using Hydrolysed FW with B. mycoides ICRI89

The current investigation used hydrolysed FW to examine the effectiveness of *B. mycoides* ICRI89 in the formation of PHB. *B. mycoides* ICRI89 was isolated locally from the soil and was investigated for PHB production in our previous research [11]. The PHB generation was carried out in 2000 mL Erlenmeyer flasks with a 1000 mL working volume. Different hydrolysed FW concentrations (5, 10, 15, 20, and 25% (*v*/*v*)) in distilled water were investigated to select the optimum PHB productivity. The flasks were then incubated at a pH of 7.0 with an inoculum concentration of 5% (*v*/*v*). For 120 h, the incubation temperature was adjusted to 35 °C at 160 rpm. Samples were collected every 24 h for the detection of biomass and PHB content within bacterial cells [32].

To extract the generated polyester, the fermentation medium was subjected to centrifugation at 4 °C in a cooling centrifuge at 4500 rpm for 15 min. The resulting cell pellets were freeze-dried, and then the bacterial cell wall was disrupted with treatment with hot acetone at 50 °C for 20 min. The suspension was centrifuged at 4500 rpm for 15 min to remove excess acetone before drying. To dissolve PHB, chloroform was used at 37 °C for 48 h while shaking at 160 rpm. Finally, the polymer was precipitated using a mixture of cold methanol and water at a ratio of 7:3 (*v*/*v*) [11].

### 2.6. Preparation of PHB/PMMA Blends

Different mass ratios (*w*/*w*) of PHB and PMMA were blended PHB/PMMA as follows: 1:1, 2:1, 3:1, 1:2 and 1:3. The polymeric pellets were melt-blended in a Haake 5000 Rheomix (Thermo Scientific, Bremen, Germany) internal mixer with two rotors at 205 °C and 55 rpm for 6 min. The polymeric blends were then poured onto Teflon plates and allowed to remain at room temperature to form films for further analysis.

### 2.7. Mechanical Properties of PHB/PMMA Blends

A digital micrometre (Mitutoyo 293-831-30, Mitutoyo, Andover, UK) was utilised to determine the film thickness. Electromechanical testing equipment (Zwick/Roell Z005, Ulm, Germany) was used to measure the tensile strength (TS) and the elongation at break (EB) for each sample at room temperature. The samples were cut into bone-shaped pieces with dimensions of 4.75 mm × 22.25 mm, using a crosshead rate of 5 mm/min [33].

### 2.8. Dicumyl Peroxide (DCP) Crosslinking

**Figure 1:**
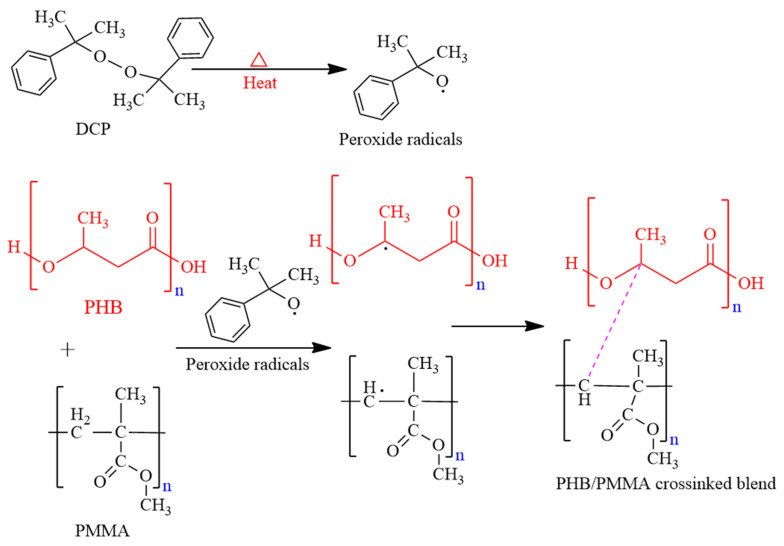
Schematic illustration of PHB and PMMA cross-inking via the formation of DCP peroxide radicals.

To investigate the effect of DCP crosslinking on the physical strength of the developed blends (Figure 1), the crosslinker was added to the melted polymeric blend with different concentrations (0.1–0.5 wt%), as previously mentioned in Section 2.6. The resulting TS, EB, and film thickness after the crosslinking were determined and compared.

### 2.9. Chemical Structure of the Generated PHB/PMMA Blend

#### FTIR

The chemical structure of the generated blends was detected through FTIR analysis. The experiment was carried out in the 400–4000 cm^−1^ range utilising an FTIR Nicolet 6700 spectrophotometer and OMNIC 3.2 software (Thermo Scientific Products: Riviera Beach, FL, USA) [34].

### 2.10. Thermal Characteristics of the Developed Crosslinked Polymeric Matrix

#### 2.10.1. Thermogravimetric Analysis, TGA

Thermogravimetric analysis (TGA) was performed using a Mettler Toledo TGA/DSC 2 STAR System (Mettler Toledo, Giessen, Germany) in ramp mode heated from 0 to 600 °C, with a heating rate of 10 °C/min and a flow rate of 20 mL/min of N2. The derivative TG (DTG) curve was created to describe the weight loss rate as a function of temperature [33].

#### 2.10.2. Differential Scanning Calorimetry (DSC)

Differential scanning calorimetry (DSC) analysis using the Mettler Toledo1 series (Ohio, OH, USA) was employed to measure the melting point (Tm) and glass-transition temperatures (Tg) of polymer samples in an N2 atmosphere at a flow rate of 20 mL/min. A 3.0 mg amount of each sample was placed in an aluminium pan and heated at a rate of 10 °C/min from −10 °C to 200 °C for DSC analysis. The point of inflection in the DSC curve between the onset and offset temperatures reveals the glass transition temperature and the melting point, which was reported as the endothermic event’s peak temperature [35].

### 2.11. Degradation Studies of the DCP-PHB/PMMA Blend

#### 2.11.1. Soil Degradation

Soil-degradation studies were carried out in a laboratory setting at a temperature of 25 °C using soil beds containing 2% humus content, 22–24% water content, and a pH of 6.5, which were kept in glass containers. These soil beds were microbially active. Samples of PHB and DCP-PHB/PMMA blend films, weighing 100 mg and with a thickness of 0.3–0.4 mm, were immersed in the soil at a depth of 15 cm for 60 days with an interval of 5 days. After the specified time period, the test pieces were removed from the containers, washed, cleaned using sonication, dried, and weighed [36]. As is typical, the degradation of the polymeric samples was evaluated and compared in percentage weight loss, utilising the following equation:

$$Degradation\;(\%)=\frac{Initial\;weight\;of\;polymer\;sample-Weight\;of\;the\;polymer\;sample\;after\;degradation}{Initial\;weight\;of\;polymer\;sample}\times 100$$

#### 2.11.2. Enzymatic Degradation

The polymeric films were placed in a solution of PBS (phosphate-buffered saline, pH 7.4) that contained 0.1 g/L of Lipase B *C. antarctica* immobilised on Immobead 150, recombinant from *Aspergillus oryzae* (Sigma-Aldrich, St. Louis, MI, USA) and with an enzyme activity of ≥1800 U/g. The degradation process was conducted on 6-well plates, where the PBS solution containing the lipase was changed every five days to prevent any changes in the lipase concentration due to potential water evaporation during degradation. The samples were periodically taken out of the solution, washed with distilled water three times, and then dried using a vacuum before weight determination over 50 days [37].

### 2.12. Filament Fabrication and SEM Analysis

The DCP-PHB/PMMA matrix granules underwent a 2 h drying process at 60 °C using an AIRID polymer dryer (3devo, Utrecht, The Netherlands). Subsequently, the dried material was fed into the hopper of a twin-screw extruder filament maker (3devo, Utrecht, The Netherlands). The extruder temperature profiles were adjusted to 170, 180, 180, and 195 °C while maintaining a screw speed of 50–60 rpm. The resulting extrudate was cooled through a dual air-cooling system to produce a filament with a diameter of 1.75 mm [38]. The morphology of the generated DCP-PHB/PMMA filament was analysed using a Scanning Electron Microscope (SEM) (HITACHI S-4700, Tokyo, Japan). The microscope used an accelerating voltage of 25 kV and could achieve a resolution of 1.2 nm. Prior to examination, the samples were coated with gold for 30 s using LUXOR^Au^, Gold Sputter Coater (Nanoscience Instruments Inc., Phoenix, AZ, USA) [39].

### 2.13. Three-Dimensional Printing Models

To create 3D models using the DCP-PHB/PMMA matrix, a RAISE3D E2 3D printer (Raise3D, Irvine, CA, USA) was used. A 3D “key of life” model with dimensions of 55 mm length × 25 mm width × 4 mm height according to ASTM D790 was constructed using the ideaMaker version 4.3.2 software. The 3D printing parameters were as follows: print nozzle diameter, 0.4 mm; nozzle temperature, 200 °C; bed temperature, 55 °C; print speed, 25 mm/s; print infill density, 100%; line infill pattern; and layer height, 0.2 mm [40].

### 2.14. Statistical Analysis

The tests were conducted three times each, and the results are presented as the average values ± the standard deviation (SD). The graphs obtained were analysed using Prism 7 software from Graphpad, Boston, MA, USA. To perform comparisons between the different groups that were studied, the Analysis of Variance (ANOVA) test was used, followed by Tukey’s Multiple Comparison test. The level of significance for the results was determined to be 5%.

## 3. Results and Discussion

### 3.1. Glucose Liberation in Response to FW Hydrolysis

It is acknowledged that the cost of PHB production has been curtailing its massive industrial implementation to obtain sustainable, eco-friendly products. One of the most impressive measures to overcome this issue is reducing the carbon source expenditure [41]. In this sense, valorising FW has been considered as an upcoming strategy to support microbial fermentation by sacrificing its starch and cellulosic content. Since PHB bacterial producers usually have limited access to simple glucose units from such complex raw materials, the pretreatment of organic FW has become paramount for better nutrient uptake via microbial factories. Sonication is frequently used in biological and chemical processes such as waste treatment, pollutant breakdown in soil, material synthesis, the alteration of the functional characteristics of dairy proteins, and surface cleaning. Various wastes have been pretreated with ultrasound to make them more susceptible to later enzymatic hydrolysis [42,43]. It has been proposed that FW particles may be physically disintegrated with the aid of sonication to improve the subsequent saccharification [29]. On the other hand, ethanol organosolv pretreatment has been shown to be highly efficient in the removal of lignin and xylan fractions from complex organic FW, paving the way for a more effective enzymatic attack and hence an elevated glucose yield [44]. In the current investigation, the efficiency of two pretreatment approaches, ultrasonication and ethanol organosolv, were compared for better subsequent saccharification through enzymatic hydrolysis via cellulase, α-amylase, and glucoamylase.

**Figure 2:**
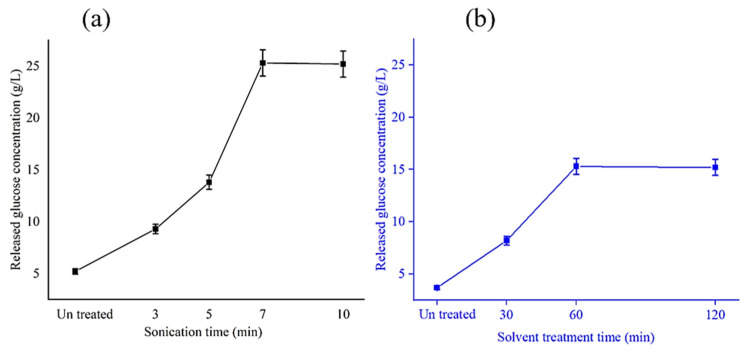
Liberated glucose content (g/L) from FW (50 g) via enzymatic hydrolysis as a function of (**a**) sonication duration (20 kHz, 0.8 W/mL at 25 °C) and (**b**) ethanolic organosolv pretreatment timing (80% *v*/*v*, 120 °C).

In Figure 2a, it is apparent that the time of the sonication process (20 kHz) was highly influential on the glucose concentration released after the enzymatic hydrolysis process. The sonication time of 7 min at 25 °C caused the reduction in the raw material particle size and the disintegration of the FW mass, giving the enzymatic molecules better access to saccharification, reaching 25.3 ± 0.22 g/L per 50 gFW. Exceeding the sonication time of 10 min had no significant effect (*p* > 0.05) on glucose liberation after the enzymatic action. Dan et al. successfully hydrolysed campus canteen FW through ultrasonication for 10 min prior to raw material saccharification for durable PHA synthesis via *Rhodopseudomonas palustris* [45]. Furthermore, a recent investigation has reported remarkable starch and cellulose solubilisation and saccharification using sonication pretreatment before the hydrolysis of enzyme mixtures, yielding 17.16 g/100 g of household FW. Also, our results were in line with those of Li et al., where ultrasonication (20 KHz, 0.8 W/mL at 20 °C) for 5 min could boost the glucose release levels from FW after glucoamylase hydrolysis compared to their untreated counterparts [29].

In another avenue, one of the most popular physical pretreatment methods that has fewer environmental effects and lower operational costs is thermal pretreatment using water and organic solvents at 100–240 °C [46]. In our study, after the enzymatic hydrolysis process, such a pretreatment approach resulted in 15.3 ± 0.13 g/L glucose after 60 min at 120 °C compared with 3.7 ± 0.05 g/L for the untreated FW (Figure 2b). Our findings were consistent with and even higher than those of Sulbarán-Rangel et al., who hydrolysed FW using 50% (*v*/*v*) ethanol to water at 175 °C for 2.5 h, releasing a glucose concentration of 6.23 ± 0.34 g/L [47]. However, as reported in a study by Yin et al. [48], high hydrothermal FW treatment led to the production of toxins that inhibited microorganisms for fermentation. Furthermore, our organosolv records were much lower than those of ultrasonication. For this reason, the ultrasonication pretreatment should be selected side by side with the enzymatic mixture hydrolysis for the generation of reliable feedstock for PHA fermentation.

### 3.2. PHB Generation from Hydrolysed FW via B. mycoides ICRI89

**Figure 3:**
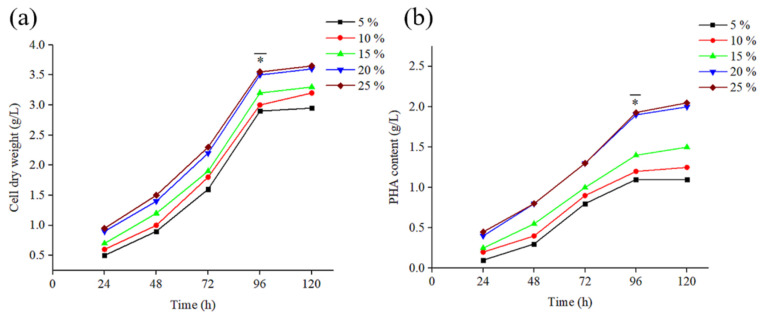
(**a**) Cell dry weight (CDW) and (**b**) PHB content (g/L) generated from *B. mycoides* ICRI89 using different FW hydrolysate percentages at different incubation time intervals over the batch fermentation process. Cultures were incubated at 35 °C under shaking conditions (160 rpm). * *p* > 0.05.

In this study, hydrolysed FW was selected as a PHB production medium for *B. mycoides* ICRI89. Ultrasonically pretreated FW was involved in two steps of enzymatic hydrolysis, which were cellulase, α-amylase, and glucoamylase. The dry weight of *B. mycoides* ICRI89 cells (CDW) was estimated using five different concentrations of hydrolysate of FW over 120 h (Figure 3a). It was clear that CDW was directly proportional to FW hydrolysate content in the fermentation medium, reaching aCDW of 3.56 ± 0.11 g/L after 120 h of incubation. A similar pattern was observed for the content of PHB, where 25% FW hydrolysate was optimal the highest accumulation of PHB of 2.11 ± 0.06 g/L after 120 h, representing a percentage of productivity of 59.3 wt% (Figure 3b). We postulate that the high percentage of FW hydrolysate provided *B. mycoides* ICRI89 cells with not only high carbon content but also vital nutrients, which were essential for PHB build-up in the bacterial inclusion bodies. On the other hand, 5% FW hydrolysate medium was the poorest in terms of glucose and nutrient supplementation. Therefore, a low PHB of 1.1 ± 0.02 g/L was accumulated at the end of the fermentation period. Another reason a high yield of PHB was obtained after 96 h is that *B. mycoides* ICRI89 factories had already entered the stationary phase, when PHB generation reaches its peak levels [34,49]. From 96 to 120 h of incubation, *B. mycoides* ICRI89 did not generate significant cell populations or PHB yield (*p* > 0.05) for all hydrolysate percentages.

The production of PHB by *B. mycoides* ICRI89 has recently been investigated in our lab using cellulase-hydrolysed cardboard as an affordable fermentation feedstock. However, when cardboard hydrolysate was exploited as the sole carbon source, a lower PHB concentration of 0.4 ± 0.1 g/L was recorded compared to the current investigation [11]. This could be because complex FW is usually much more nutritive than paper waste. To illustrate, FW includes numerous essential elements such as carbon, nitrogen and phosphorus, which are vital for bacterial cell biomass and PHB build-up. In this sense, there was no need for the additional supplementation of nutrients to batch fermentation, which is optimal for economical scaling up [50].

**Figure 4:**
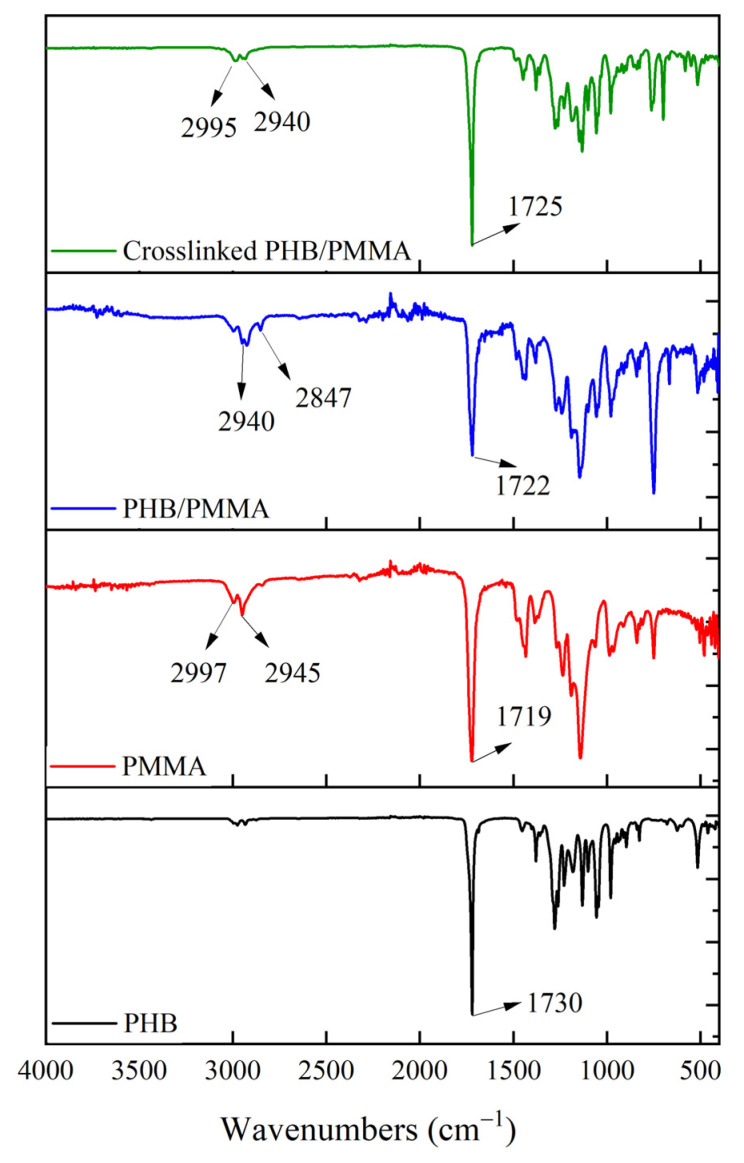
The chemical structure of PHB produced *B. mycoides* ICRI89, standard PMMA, PHB/PMMA, and DCP-PHB/PMMA blends.

The extracted and purified polymer was investigated for its chemical structure using FTIR. A high absorption peak was visible around 1120 cm^−1^, which was associated with a saturated ester bond of (CO-) groups. Furthermore, a stretching of the methyl (CH_3_) group was detected at 1345 and 1425 cm^−1^ (Figure 4). More importantly, the peaks at 3350, 2920, and 1730 cm^−1^ were indicative of hydroxyl (OH), methine (=CH-), and carbonyl (C=O), groups, respectively. The common homopolymer of PHAs, known as PHB, was distinguished by the carbonyl group’s (C=O) peak absorbance (Figure 4) [11].

### 3.3. Effect of Different Polymeric Ratios on PHB/PMMA Blends’ Mechanical Properties

The influence of varying ratios of PHB and PMMA was investigated in terms of film thickness, TS, and EB. The neat PHB and PMMA films had TS values of 30.2 ± 0.06 and 59.4 ± 0.1 MPa, as well as EB records of 7.6 ± 0.01 and 5.5 ± 0.02%, respectively. Our records were comparable with those of previous reports [51,52]. Furthermore, it is apparent from Table 1 that TS increased smoothly by enhancing PHB content. Interestingly, TS values increased dramatically with high PMMA contents, reaching 66.2 ± 0.06 MPa for the PHB/PMMA (1:3) blend. This phenomenon is consistent with a previous investigation by Wan and Zhang, where PLA/PMMA composites possessed the most satisfactory TS, owing to the formation of interpenetrating networks after enhancing the acrylic content, ameliorating the polymer resistance to the pulling forces [53]. Considering the EB, the values were directly proportional to the PHB content; however, at the highest level (PHB/PMMA (3:1)), EB slightly dropped to 6.3 ± 0.04%. This could be related to the brittle nature of PHB as a highly known shortcoming [54]. The same behaviour was also observed in the other ratios, where PHB/PMMA (1:3) witnessed a slight decrease in EB value (8.9 ± 0.01%) as compared to the highest record of 9.8 ± 0.11% for PHB/PMMA (1:2). The considerable increase in EB values could be indicative of the good compatibility between both polymers, reflecting the positive role of PMMA in improving EB values [53]. On the other hand, the decline in EB in highly acrylate blends (PHB/PMMA (1:3)) could be due to the rigid structure of PMMA that makes the blend more vulnerable to breakage [55]. Since the PHB/PMMA (1:2) blend possessed the highest TS and EB records, it should be selected for DCP crosslinking studies.

### 3.4. DCP Crosslinked Blends

Melt-blending was used to create the PHB/PMMA blend in the presence of DCP. DCP can initiate free radicals on both PHB and PMMA chains through a hydrogen-absorption process at high temperatures [56], and as a result, grafting occurs at the interface of the PHB/PMMA blends via a combination of free radicals. Hence, a reliable crosslinked polymeric blend of the DCP-PHB/PMMA network can be obtained. There was a positive correlation between the mechanical properties (TS and EB) of DCP-PHB/PMMA (1:2 blend) and the content of DCP at a concentration of 0.3 wt%, reaching 78.6 ± 0.06 MPa and 10.9 ± 0.12%, respectively (Table 2). However, a further increase in the DCP content to 0.4 and 0.5 by weight resulted in a decrease in the TS and EB values. The observable rise in TS and EB at 0.3 wt% could be correlated to the gel-formation ability of the resultant DCP-PHB/PMMA blend, which is crucial for the processability of the final matrix in various applications [56]. It is worth mentioning that the inclusion of DCP might have enhanced the TS of PHB blends by creating a free radical interaction between the main composing polymers, resulting in partially branched and/or crosslinked structures. Furthermore, the addition of DCP had little effect on the surface colour of the blends [57]. On the other hand, the relative decline in TS and EB values at 0.4 and 0.5 wt% of DCP could be due to an extensive crosslinking, bringing about a high possibility of chain slippage [58]. Our findings agree with those of Semba et al. [59], who also recommended DCP introduction to boost the mechanical properties of PLA/PCL films through radical formation.

### 3.5. Chemical Characterisation of PHB/PMMA Film

The FTIR spectra of PMMA showed a distinct, intense peak at 1719 cm^−1^, which corresponded to the ester carbonyl group of the polymer. The large peak stretching in the range of 980–1240 cm^−1^ indicated the stretching vibration of the C-O (ester bond). The bending of C-H was detected between 640 and 950 cm^−1^ (Figure 4). The vibration of hydroxyl group (-OH) was detectable based on a wide peak from 2900 to 3100 cm^−1^ [52]. In the PHB/PMMA film, the band of the (C=O) group moved to 1722 cm^−1^, with reduced intensity and wider distribution, which indicated an efficient interaction between PHB and PMMA. Furthermore, C-H bending was revealed based on a large peak between 653 and 948 cm^−1^ (Figure 4). As for DCP-PHB/PMMA, the CH_3_ asymmetric stretching vibration at 2995 cm^−1^ shifted to lower wavelengths of -CH at 2940 and 2847 cm^−1^. To illustrate, the cumyloxy radicals could be broken down into acetophenone and methyl radicals, which induced CH_3_ to stretch at 2940 cm^−1^ [60]. Moreover, the molecular interaction between the two polymers triggered a modest expansion of the C=O stretching vibration at 1725 cm^−1^. The reason behind the effective PHB/PMMA crosslinking is the preferential attack of DCP on the tertiary carbon atoms of the reactive polymers, forming peroxide radicals (Figure 1). By way of conclusion, the shift in vibrational frequencies, the expansion of specific functional groups, and the formation of peroxide radicals all provide robust evidence for the occurrence of crosslinking in the DCP-PHB/PMMA system, suggesting that the two polymers chemically bind together through crosslinking reactions. Similarly, PLA/cellulose and PHB/cellulose radicals interact during the propagation and termination steps, which have been proven by previous Electron Spin Resonance (ESR) observations [61,62].

### 3.6. Thermal Analysis of the DCP-PHB/PMMA Blend as Compared to Native Polymers

**Figure 5:**
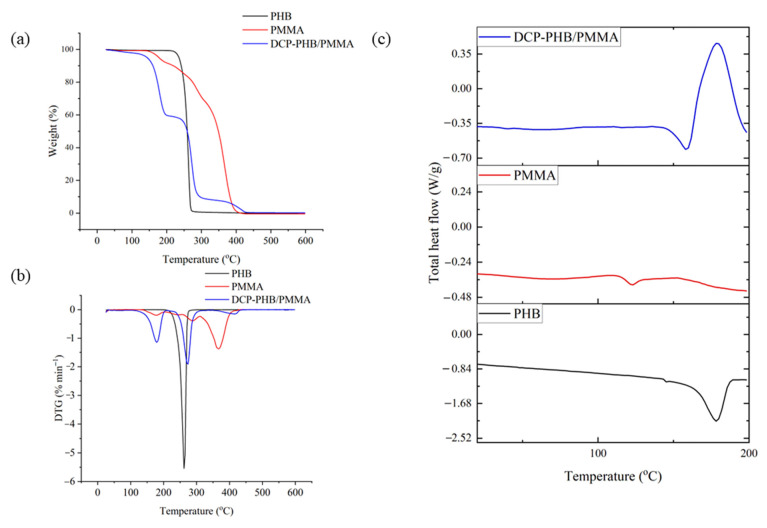
Thermal analysis of PHB, PMMA, and DCP-PMMA/PHB blend including (**a**) TGA, (**b**) DTG, and (**c**) DSC.

Figure 5a shows the TGA mass loss of neat PHB and PMMA, as well as the investigated DCP-PHB/PMMA blend. The highest degradation temperature of PHB was found to be 275 °C. The current findings are in agreement with those of Pradhan et al. [63], who reported 289 °C as the maximal degradation temperature of PHB generated by *Cupriavidus necator*. Furthermore, neat PMMA had a maximum degradation temperature of 420 °C, which could be attributed to random scission and depropagation [64]. Remarkably, the crosslinked DCP-PHB/PMMA exhibited a maximal degradation temperature of 415 °C. PHB and PMMA blending, as well as DCP crosslinking, had a positive effect on the maximum degradation temperature value and a substantial effect on the onset temperature of degradation. Thus, the thermal stability of the DCP-PHB/PMMA blend was significantly enhanced compared to neat polymers [65,66].

The peaks observed in the DTG curves (Figure 5b) indicated the thermal stability of PHB, PMMA, and the DCP-PHB/PMMA blend with respect to the temperature at which the maximum degradation rate of the polymer matrix occurs. The DCP-PHB/PMMA blend exhibited a thermal stability that was higher than PHB but lower than PMMA (Figure 5b). The DCP-PHB/PMMA exhibited a two-step weight loss corresponding to the PHB phase at 150.35–210.22 °C [67] and the PMMA phase at 262.22–315.5 °C [68]. In this sense, the degree of weight loss at each step coincided well with the composition of the blend. It is noted that the presence of DCP had a pronounced effect on the thermal decomposition temperatures of the generated blend. That is to say, degradation temperatures usually increased when the cohesive forces in the matrix increased, which were affected by the crosslinking and crystallisation degree of the reacting polymers [69].

Considering DSC measurements, PHB and PMMA had glass transition temperatures (Tg) of 6.5 and 110.0 °C, respectively. The DCP-PHB/PMMA blend had a single Tg of 25 °C that was greater than that of pure PHB, implying that the two components were completely miscible in the presence of DCP. Notably, PHB exhibited a large peak at about 178 °C, which is similar to its melting point (Tm) (Figure 5c). The inclusion of PMMA, which had a Tm of 132 °C, slowed the process of PHB chain crystallisation, bringing about a lower melting point of 158 °C and a cold crystallisation temperature (Tcc) of 197 °C for the resultant blend. This finding was extra proof for the DSC results, demonstrating that PHB and PMMA were entirely miscible after mixing [24]. With the addition of PMMA to the mixture, the cold crystallization enthalpy was observed to be lower, proving that PMMA prevented PHB from crystallization.

### 3.7. Biodegradation Profiles of the Developed DCP-PHB/PMMA Blend

#### 3.7.1. Soil Degradation

**Figure 6:**
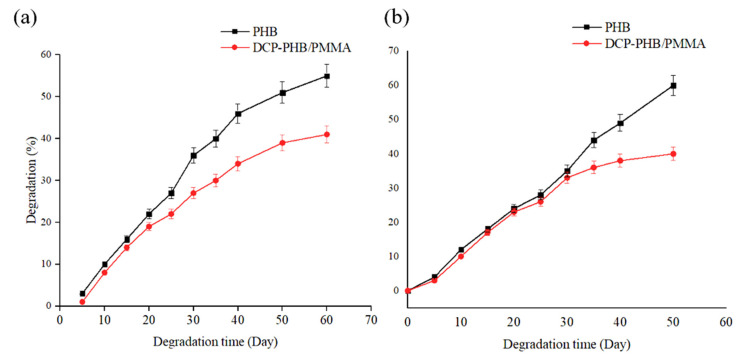
Degradation profile of PHB as a standard biodegradable polymer in comparison with the DCP-PHB/PMMA blend through (**a**) soil degradation and (**b**) enzymatic degradation.

PHB has the advantage of degrading under both aerobic and anaerobic conditions, resulting in a rapid decomposition rate and, as a result, a minimal ecological effect, especially when compared to other biopolymers. Throughout the biodegradation investigation, the microenvironment temperature was fixed at 25 °C. This temperature was within the range that mesophilic bacteria (25–40 °C) and fungi (22–30 °C) generally prefer for biodegradation. Within 60 days, the biodegradation of neat PHB and DCP-PHB/PMMA films was observed. Both films exhibited comparable biodegradation rates; however, the PHB film had higher values than those of DCP-PHB/PMMA, with almost 10.2% on day 35. On day 60, approximately 55.6% of PHB membranes were degraded versus 41.3% for the DCP-PHB/PMMA film (Figure 6a). It is noteworthy that controlling the soil beds’ conditions, such as using 2% humus content, 24% water content, and a pH of 6.5, induced a microbially active environment optimal for biodegradation. The biodegradation of PHB is usually related to the microbial enzymatic action [70]. On the contrary, PMMA is an environmentally friendly non-biodegradable polymer whose biodegradability is enhanced through blending with other biodegradable polymers. To illustrate this, the PHB component of the buried DCP-PHB/PMMA blend samples would first biodegrade through the action of various microbial communities in the soil. Oxygen can then damage the freshly created surface by producing peroxides, hydroperoxides, oxides, etc., which encourage the scission of PMMA polymeric chains into short fragments. These chains are considerably more susceptible to microbial degradation [71]. This is why the CDP-PHB/PMMA blend had biodegradation rates comparable to those of native PHB after 60 days in a controlled environment. From the previous data, the crosslinking of PHB with PMMA had a major role in commencing PMMA biodegradation in microbially active soil.

#### 3.7.2. Enzymatic Degradation

The DCP-PHB/PMMA blend was exposed to Lipase B enzyme with the aim of investigating its biodegradation profile. Lipase B from *C. antarctica* was able to degrade about 40.4% from the DCP-PHB/PMMA film in comparison with 60.2% from the PHB film over 50 days (Figure 6b). According to Sudhakar et al., lipase B has the ability to attach ester bonds existing in the biodegradable portion in the investigated blends (cellulose acetate butyrate), followed by peroxide generation, which, in turn, caused PMMA long-chain disintegration [71]. Similarly, in our study, it seems that active lipase B in a controlled environment had a key role in breaking ester bonds in the PHB backbone, where the resulting peroxides attached to PMMA chains, triggering a biodegradation percentage of 40.4%. It is worth mentioning that the biodegradation rate of lipase-treated PHB was higher than that of the buried polyester in soil by almost 19.8%. This could be related to the fact that exposure to pure enzymes is usually more rapid and effective than exposure to active cells metabolising enzymes for the generation of enzymes. Nevertheless, enzyme treatment could be extremely costly when it comes to addressing global waste-accumulation issues.

### 3.8. Fabrication of the Polymeric Filament and 3D Printing Studies

**Figure 7:**
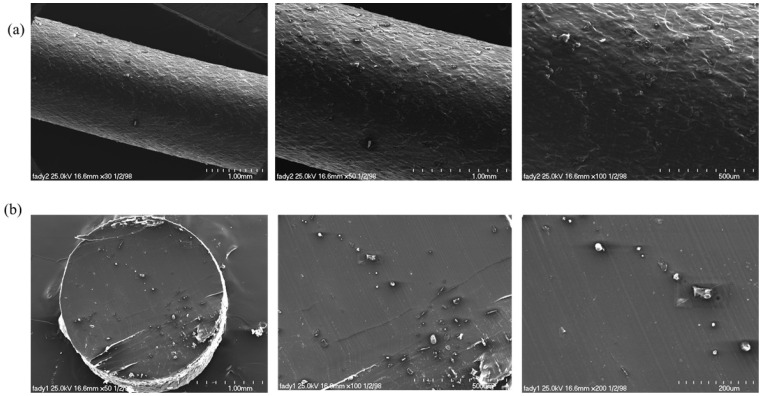
SEM morphological analysis of (**a**) surface and (**b**) cross-section of the fabricated DCP-PHB/PMMA extrudate filament.

SEM was employed to analyse the morphology of the DCP-PHB/PMMA extrudate’s filament surface and cross-sections. Normally, PHB is a semi-crystalline polymer with a high degree of crystallinity; however, when PMMA was added, it reduced the crystallinity of PHB, leading to a less ordered crystalline structure, which was also reflected by the smooth surface of the filament. This also could be due to the better compatibility of the two phases of PHB and PMMA (Figure 7a). Owing to the beneficial effect of the DCP crosslinking agent, the fracture surface of the PHB/PMMA filament revealed a homogeneous structure with good dispersion and adhesion. These findings indicate that DCP had a beneficial influence on enhancing the interfacial adhesion between the PHB and PMMA matrix, which also boosted their compatibility. SEM imaging of the filament cross-sections also exhibited a smooth surface free of air bubbles (Figure 7b), confirming that the twin-screw extrusion effectively converted the polymer crystals into an amorphous form due to the intensive mixing and torque generated throughout the extrusion process.

**Figure 8:**
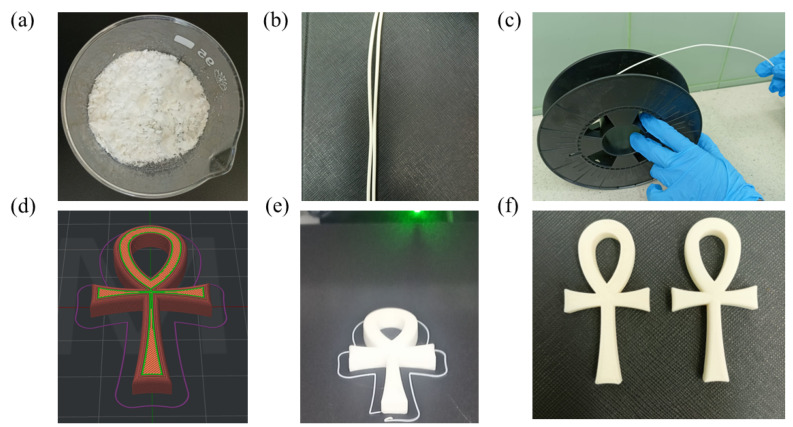
DCP-PHB/PMMA filament fabrication and 3D-printed models. (**a**) DCP-PHB/PMMA (1:2) ground matrix, (**b**) resultant filament after extrusion, (**c**) rolling the filament on a spool, (**d**) “key of life” model using ideaMaker version 4.3.2 software, and (**e**,**f**) multiple “key of life” models, which were printed for validation.

The purified and dried DCP-PHB/PMMA matrix was ground to reach a particle size of around 0.4–0.8 mm to ease the feeding process through the extruder. The polymer matrix exhibited a white powdery appearance (Figure 8a). The resultant filament had a smooth, uniform structure throughout the full length with a diameter of 1.75 mm. In addition, the filament extrusion was highly satisfactory, since the filament did not possess breaks, bubbles, or blockage (Figure 8b). The filament was flexible enough to be perfectly spooled without breakage, which could be attributed to the mild crosslinking between PHB and PMMA (Figure 8c). Furthermore, the selected “key of life” model was adjusted using ideaMaker version 4.3.2 software to provide a fill percentage of 60% and dimensions of 55 mm × 25 mm × 4 mm (Figure 8d). The printing time lasted for almost 19.2 min without extra support. The output model was identical to that of the software, with a smooth surface and white color (Figure 8e). There was no adhesion problem between the model and the magnetic plate, which made the printing process more controllable. Interestingly, multiple “key of life” models (Figure 8f) have been sustainably produced straightforwardly, opening new avenues for affordable, biodegradable, 3D-printed models with ameliorated properties for versatile applications.

## 4. Conclusions

Food waste from local restaurants was fully saccharified and adjusted in the search for an efficient and durable substrate for the biosynthesis of PHB. Ultrasonication pretreatment combined with an enzymatic mixture brought about a satisfactory release of glucose for the start of batch fermentation. Furthermore, *B. mycoides* ICRI89 achieved an intensive PHB productivity of more than 50% after 120 h when hydrolysed FW was the only feeding medium. The resultant polymer was confirmed using FTIR to be a PHB homopolymer. Upon mixing with PMMA, TS rose slightly by elevating the PHB content in the blends. However, TS increased considerably in films with a high PMMA content. PHB/PMMA (1:2) had the highest TS and EB values of 63.4 ± 0.06 MPa and 9.8 ± 0.11%, respectively, reflecting the high compatibility between both polymers. DCP was effective in creating free radicals on the tertiary carbon atom on both polymer backbones, causing crosslinking and furthermore improving the mechanical properties of the resulting blend. Adding to this, the DTG pattern of DCP-PHB/PMMA blend revealed a two-step weight loss, implying the intensive mixing of both polymers. The developed blend had a single Tg of 25 °C, a Tm of 158 °C, and a Tcc of 197 °C, which indicated the high miscibility of polymers. The fabricated film also assisted in the faster disintegration of non-biodegradable PMMA since the DCP-PHB/PMMA film possessed a biodegradation percentage of 41% in active soil compared with 55% for neat PHB. DCP-PHB/PMMA films were successfully used in constructing 3D-printed models in a simple and validated manner. Therefore, the proposed bioconversion strategy and biodegradable platforms are strongly recommended for environmental and economic aspects.

## 5. Challenges and Prospects

Since the current study proposes hydrolysed FW as a sole fermentation medium for *B. mycoides* ICRI89, PHB productivity should be further investigated using bacterial hyperproducers. For example, genetically modified strains or a consortium of multiple strains could be optimised for better yield. Furthermore, several attempts have been made to reduce the total cost of large-scale implementation, such as the expenditure of carbon sources. Furthermore, developing more efficient and environmentally friendly techniques for separating PHB from microbial biomass can significantly reduce the chemical and energy-intensive steps. Techniques such as solvent extraction, enzyme-based methods, or physical separation techniques like centrifugation or filtration can be explored. Nevertheless, sterilisation techniques such as autoclaving and filtration would be another barrier facing massive generation. To amend this, extremophilic strains such as halophils or psychrophiles could be exploited to tolerate extreme conditions, dispensing the costly traditional approaches. To remove the barrier of the cost of enzymes, there would be more investigations on two-step fermentation strategies consisting of microbial enzyme generation and then PHB hyperproduction for elevated yields. Regarding the biomaterial, more experimentation could be conducted to investigate the printability of other PHB blends with improved properties and easy processability for commercialisation.

## Figures and Tables

**Table 1:** Effect of different polymeric ratios (*w*/*w*) on PHB/PMMA blends mechanical properties.

	Film Thickness (mm)	TS (MPa)	EB (%)
PHB	0.24 ± 0.12 ^a^	30.2 ± 0.06 ^a^	7.6 ± 0.01 ^a^
PMMA	0.25 ± 0.23 ^a^	59.4 ± 0.1 ^b^	5.5 ±0.02 ^b^
PHB/PMMA (1:1)	0.24 ± 0.16 ^a^	39.2 ± 0.11 ^c^	8.2 ± 0.07 ^c^
PHB/PMMA (2:1)	0.24 ± 0.07 ^a^	41.7 ± 0.06 ^d^	9.4 ± 0.02 ^c^
PHB/PMMA (3:1)	0.26 ± 0.04 ^b^	42.8 ± 0.12 ^d^	6.3 ± 0.04 ^d^
PHB/PMMA (1:2)	0.27 ± 0.09 ^b^	63.4 ± 0.06 ^e^	9.8 ± 0.11 ^e^
PHB/PMMA (1:3)	0.25 ± 0.06 ^c^	66.2 ± 0.06 ^e^	8.9 ± 0.01 ^d^

TS and EB are tensile strength and elongation at break. Superscripts with different letters in the same column mean significance (*p* < 0.05).

**Table 2:** Effect of DCP content on the mechanical properties of the generated blends.

	PHB/PMMA (1:2 *w*/*w*)		
DCP Concentration (wt%)	Film Thickness (mm)	TS (MPa)	EB (%)
0.1	0.27 ± 0.12 ^a^	67.8 ± 0.12 ^a^	9.9 ± 0.04 ^a^
0.2	0.26 ± 0.07 ^a^	73.1 ± 0.01 ^a^	10.2 ± 0.17 ^a^
0.3	0.25 ± 0.13 ^a^	78.6 ± 0.06 ^b^	10.9 ± 0.12 ^b^
0.4	0.27 ± 0.03 ^b^	72.8 ± 0.02 ^c^	8.2 ± 0.1 ^c^
0.5	0.26 ± 0.05 ^b^	66.4 ± 0.1 ^d^	7.3 ± 0.03 ^d^

TS and EB are tensile strength and elongation at break. Superscripts with different letters in the same column mean significance (*p* < 0.05).

## Data Availability

The data presented in this study are available on request from the corresponding author.
