# Correction: Rofeal et al. Sustainable Polyhydroxyalkanoate Production from Food Waste via *Bacillus mycoides* ICRI89: Enhanced 3D Printing with Poly (Methyl Methacrylate) Blend. *Polymers* 2023, *15*, 4173

**DOI:** 10.3390/polym18010102

**Published:** 2025-12-30

**Authors:** Marian Rofeal, Fady Abdelmalek, Joanna Pietrasik

**Affiliations:** 1 International Center for Research on Innovative Biobased Materials (ICRI-BioM)—International Research Agenda, Lodz University of Technology, Zeromskiego 116, 90-924 Lodz, Poland; 2 Department of Botany and Microbiology, Faculty of Science, Alexandria University, Alexandria 21521, Egypt; 3 Chemical Engineering Department, Polytechnique Montreal, Montreal, QC H3T 1J4, Canada; 4 Department of Engineering Physics, Polytechnique Montreal, Montreal, QC H3T 1J4, Canada; 5 Faculty of Chemistry, Institute of Polymer and Dye Technology, Lodz University of Technology, Stefanowskiego 16, 90-537 Lodz, Poland; joanna.pietrasik@p.lodz.pl

## Error in Figure

In the original publication [1], there was a mistake in Figure 7 as published. Some structural features appeared differently or were partially lost due to sample deformation under the electron beam during SEM imaging. The corrected Figure 7 appears below. 

**Figure 7:**
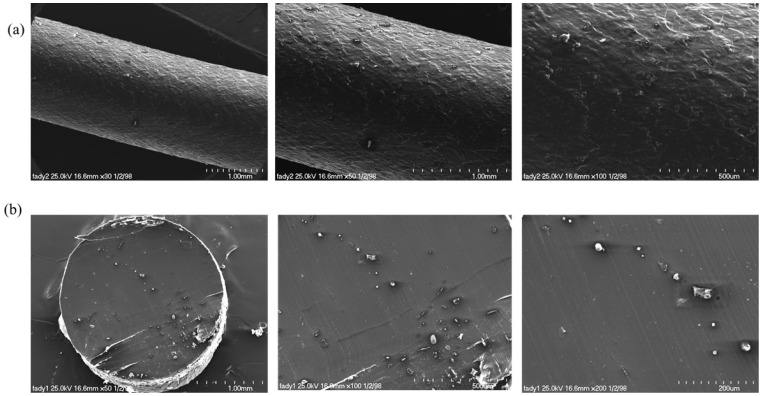
SEM morphological analysis of (**a**) surface and (**b**) cross-section of the fabricated DCP-PHB/PMMA extrudate filament.

This correction was approved by the Academic Editor. The original publication has also been updated. The authors state that the scientific conclusions are unaffected.
